# Human small intestinal infection by SARS-CoV-2 is characterized by a mucosal infiltration with activated CD8^+^ T cells

**DOI:** 10.1038/s41385-021-00437-z

**Published:** 2021-08-21

**Authors:** Malte Lehmann, Kristina Allers, Claudia Heldt, Jenny Meinhardt, Franziska Schmidt, Yasmina Rodriguez-Sillke, Désirée Kunkel, Michael Schumann, Chotima Böttcher, Christiane Stahl-Hennig, Sefer Elezkurtaj, Christian Bojarski, Helena Radbruch, Victor M. Corman, Thomas Schneider, Christoph Loddenkemper, Verena Moos, Carl Weidinger, Anja A. Kühl, Britta Siegmund

**Affiliations:** 1 Medical Department, Division of Gastroenterology, Infectious Diseases and Rheumatology, Charité - Universitätsmedizin Berlin, corporate member of Freie Universität Berlin, Humboldt-Universität zu Berlin, Campus Benjamin Franklin, Hindenburgdamm 30, 12200 Berlin, Germany; 2 Department of Neuropathology, Charité – Universitätsmedizin Berlin, corporate member of Freie Universität Berlin, Humboldt-Universität zu Berlin, Berlin, Germany; 3 Flow & Mass Cytometry Core Facility, Berlin Institute of Health at Charité - Universitä̈tsmedizin Berlin, Berlin, Germany; 4 Klinik für Psychiatrie und Psychotherapie, Campus Mitte, Charité – Universitätsmedizin Berlin, corporate member of Freie Universität Berlin, Humboldt-Universität zu Berlin, Berlin, Germany; 5 German Primate Center, 37077 Göttingen, Germany; 6 Institute of Pathology, Charité – Universitätsmedizin Berlin, corporate member of Freie Universität Berlin, Humboldt-Universität zu Berlin, Berlin, Germany; 7 The Transregio 241 IBDome Consortium, Charité – Universitätsmedizin Berlin, corporate member of Freie Universität Berlin, Humboldt-Universität zu Berlin, Berlin, Germany; 8 Institute of Virology and German Centre for Infection Research, Charité – Universitätsmedizin Berlin, corporate member of Freie Universität Berlin, Humboldt-Universität zu Berlin, Berlin, Germany; 9 Berlin Institute of Health Charité Clinician Scientist Program, Charité – Universitätsmedizin Berlin, corporate member of Freie Universität Berlin, Humboldt-Universität zu Berlin, Berlin, Germany; 10 PathoTres, Gemeinschaftspraxis für Pathologie und Neuropathologie, Teltowkanalstr. 2, 12247 Berlin, Germany; 11 iPATH.Berlin, Campus Benjamin Franklin, Charité – Universitätsmedizin Berlin, corporate member of Freie Universität Berlin, Humboldt-Universität zu Berlin, Berlin, Germany

## Abstract

The SARS-CoV-2 pandemic has so far claimed over three and a half million lives worldwide. Though the SARS-CoV-2 mediated disease COVID-19 has first been characterized by an infection of the upper airways and the lung, recent evidence suggests a complex disease including gastrointestinal symptoms. Even if a direct viral tropism of intestinal cells has recently been demonstrated, it remains unclear, whether gastrointestinal symptoms are caused by direct infection of the gastrointestinal tract by SARS-CoV-2 or whether they are a consequence of a systemic immune activation and subsequent modulation of the mucosal immune system. To better understand the cause of intestinal symptoms we analyzed biopsies of the small intestine from SARS-CoV-2 infected individuals. Applying qRT-PCR and immunohistochemistry, we detected SARS-CoV-2 RNA and nucleocapsid protein in duodenal mucosa. In addition, applying imaging mass cytometry and immunohistochemistry, we identified histomorphological changes of the epithelium, which were characterized by an accumulation of activated intraepithelial CD8^+^ T cells as well as epithelial apoptosis and subsequent regenerative proliferation in the small intestine of COVID-19 patients. In summary, our findings indicate that intraepithelial CD8^+^ T cells are activated upon infection of intestinal epithelial cells with SARS-CoV-2, providing one possible explanation for gastrointestinal symptoms associated with COVID-19.

## Introduction

In December 2019, the disease known as Coronavirus Disease 2019 (COVID-19) caused by severe acute respiratory syndrome coronavirus 2 (SARS-CoV-2) was reported for the first time and has by now been claimed over three and a half million lives around the world^1^. COVID-19 is mainly characterized by symptoms caused by a viral infection of the upper airways and the lung such as fever, cough, dyspnea, anosmia, and fatigue^2^. However, an increasing number of reports indicate that COVID-19 is more of systemic nature, including cardiovascular, haematological, renal, neurologic, and dermatologic as well as gastrointestinal manifestations^3^. To date it remains unknown, whether these effects are caused by direct SARS-CoV-2 infection of the respective tissues^4–7^, indirectly by the excessive release of cytokines^8^, by thromboembolic effects impairing microcirculation^3^, or by a combination of all mentioned complications.

Gastrointestinal involvement, likely including virus replication, has already been reported for other coronaviruses, including SARS-CoV^9^, the Middle East Respiratory Syndrome (MERS)^10^ and the four endemic human Coronaviruses^11^. In a recent meta-analysis, 17.6% of patients suffering from COVID-19 reported gastrointestinal symptoms such as loss of appetite, nausea/vomiting, diarrhea, and/or abdominal pain/discomfort^12^. In the same study, the pooled prevalence of stool samples positive for SARS-CoV-2 RNA was 48.1%, of which 70.3% of the samples remained positive for viral RNA even when respiratory specimens were tested negative for viral RNA^12^. Recently, SARS-CoV-2 RNA was not only shown to be present in feces but also in esophageal, gastric, duodenal, ileal, and rectal biopsies of SARS-CoV-2 infected individuals^13^. It has been demonstrated that SARS-CoV-2 replicates in the colonic cell line C2BBe1 having a brush border^14^. Lamers and colleagues revealed that SARS-CoV-2 was not only able to enter but also to replicate within cells of human small intestinal organoids derived from primary human small intestinal epithelial stem cells^15^. This was promoted by the serine proteases transmembrane protease serine subtype (TMPRSS) 2 and TMPRSS4 which facilitate virus entry into intestinal organoid host cells^16^. This is in line with experiments conducted by Sia and colleagues, who found expression of SARS-CoV-2 N-protein in the intestine of golden hamsters after exposure to the virus^17^. Additionally, viral nucleocapsid protein could be detected in a patient suffering from COVID-19 in the cytoplasm of gastric duodenal and rectal but not esophageal epithelial cells^18^. A recently published study by Chu et al. showed viral replication ex vivo in surgically removed intestinal tissue but not in kidney or liver tissue^19^. In addition, COVID-19 patients were proven to have elevated plasma levels of LPS-binding protein indicating gut leakage and CCL25, a gut homing biomarker^20^.

For entering a cell, SARS-CoV-2 uses the angiotensin-converting enzyme 2 (ACE2) and TMPRSS2. Priming of the viral spike (S) protein by TMPRSS2 is followed by its binding to the receptor ACE2^21^. This receptor is not exclusively found on cells of the upper airway^22,23^, but is also expressed on small intestinal enterocytes^24,25^. However, ACE2 expression could not be found on goblet or intestinal immune cells^24^. The expression of ACE2 on small intestinal enterocytes was even higher than on cells of the upper airways^26^, suggesting that nasal mucus containing infectious virus particles is probably digested and transports the virus into the human gastrointestinal system.

Data on the histomorphologic changes due to an infection of the intestinal mucosa by SARS-CoV-2 is scarce. In this study, we present data on the presence of SARS-CoV-2 in the human small intestine and characterize the immunological changes in the duodenal mucosa by imaging mass cytometry (IMC) and immunohistochemistry.

## Results

### Small intestinal reaction in COVID-19 patients

**Fig. 1:**
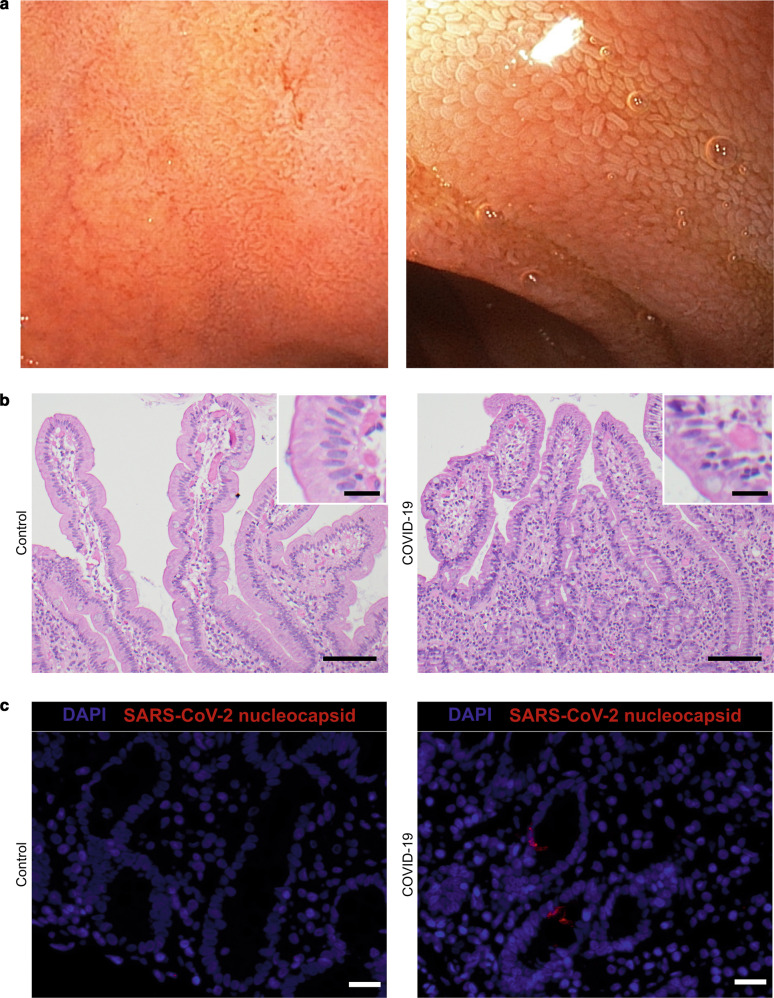
Duodenal biopsies of COVID-19 patients show histomorphologic changes. **a** Representative esophagogastroduodenoscopy images of the duodenum of COVID-19 patients. Overview (left) and close view (right). **b** H&E staining of a representative duodenal biopsy acquired from a control patient without gastrointestinal damage (left) and a patient suffering from COVID-19 (right). Inserts represent higher magnifications of the same image. Scale bars represent 100 µm in the overview and 20 µm in the magnification. **c** Representative images of SARS-CoV-2 nucleocapsid (red) and DAPI (blue) staining of duodenal biopsies from control (left) and COVID-19 (right) patients. Scale bars represent 20 µm.

To evaluate the macroscopic and histomorphologic changes in the duodenum of COVID-19 patients, duodenal biopsies were taken by esophagogastroduodenoscopy. A total of five COVID-19 patients (31–71 years, 1 female, 4 male) were included in this study (Table 1). Patients were recruited from April to June 2020 when the nasopharyngeal swab was tested positive for SARS-CoV-2 by qRT-PCR (Table 1). While three of the patients presented with gastrointestinal symptoms such as upper abdominal pain or diarrhea, respectively, at the time of admission, two of the patients reported upper abdominal pain or diarrhea, respectively, during their stay in the hospital (Table 1). No macroscopic changes were observed during esophagogastroduodenoscopy (Fig. 1a). Antibodies directed against SARS-CoV-2 (IgA and IgG) were found in one of these patients at the time of admission (Table 1). All the patients showed a moderate disease course, with no need for mechanical ventilation. The average time from onset of COVID-19 symptoms to esophagogastroduodenoscopy was 8.2 days.

**Table 1:** COVID-19 patients included in this study.

Age	Sex	GI symptoms	RT-PCR in duodenal sample	IHC	time from first symptoms to EGD	Serum IgA at time of admission	Serum IgG at time of admission	plumped villae / higher IEL	Max. Serum Ferritin (µg/l)	Max. serum IL-6 (ng/l)
33	M	Upper abd. pain^2^	−	−	9	−	−	+	1659.1	38.1
71	M	Diarrhea^1^	−	−	4	+	+	+	423.8	122.6
70	F	Diarrhea^1^	+	+	11	−	−	+	245.9	34.3
48	M	Upper abd. pain^2^	+	+	8–14	−	−	−	1784.9	30.5
31	M	Diarrhea^1^	−	−	6	na	na	+	2734.0	16.1

Patients with gastrointestinal symptoms (diarrhea, abdominal pain or discomfort, nausea/vomiting) were tested for anti-SARS-CoV-2 IgA and IgG in the serum at the time of admission. Maximum serum ferritin and serum IL-6 represent the maximum ferritin and IL-6 serum concentration measured during the time of hospitalization. *GI* gastrointestinal, *EGD* esophagogastroduodenoscopy, *IEL* intraepithelial lymphocytes, *qRT-PCT* quantitative RT-PCR, *na* not available, *abd.* abdominal, *IHC* immunohistochemistry for SARS-CoV-2 nucleocapsid; ^1^patients presented with GI symptoms at the time of admission; ^2^Patients developed GI symptoms during their stay in the hospital.

The histomorphologic analysis of the duodenal mucosa revealed in four out of five COVID-19 patients villous blunting and an increase in intraepithelial lymphocytes (IEL) (Fig. 1b and Suppl. Figure 1). Biopsies of two patients were positive for viral RNA (Table 1). SARS-CoV-2 concentrations in these two samples were 1.53 and 3.88 Log10 SARS-CoV-2 RNA copies/10,000 cells. Using an antibody directed against SARS/SARS-CoV-2 nucleocapsid, viral nucleocapsid protein was detected in the epithelium of those patients, who were tested positive for viral RNA (Fig. 1c and Table 1). While specific staining was verified in Vero cells infected with SARS-CoV-2 for 48 h (Suppl. Figure 1B), the staining of the duodenal biopsies was weak and scattered (Fig. 1c).

Collectively, we could show that the mucosa of the small intestine is potentially susceptible to infection by SARS-CoV-2. While macroscopic changes were not detectable, histomorphologic changes were overt, indicating a local reaction characterized by an increased infiltration with IELs.

### Small intestinal reaction to SARS-CoV-2 is characterized by an epithelial infiltration of activated intraepithelial CD8^+^ T cells

To further characterize the histomorphologic changes, IMC was applied for deep immunophenotyping. IMC enables the simultaneous imaging of up to 40 markers at a resolution of 1 µm^2^ employing isotope-tagged antibodies^27^. The antibody cocktail used in this study included 25 antibodies directed against proteins expressed by immune cells and non-hematopoietic cells as well as histone H3, a chromatin protein. Additionally, iridium was used as a nuclear marker intercalating into DNA (Suppl. Table 2).

**Fig. 2:**
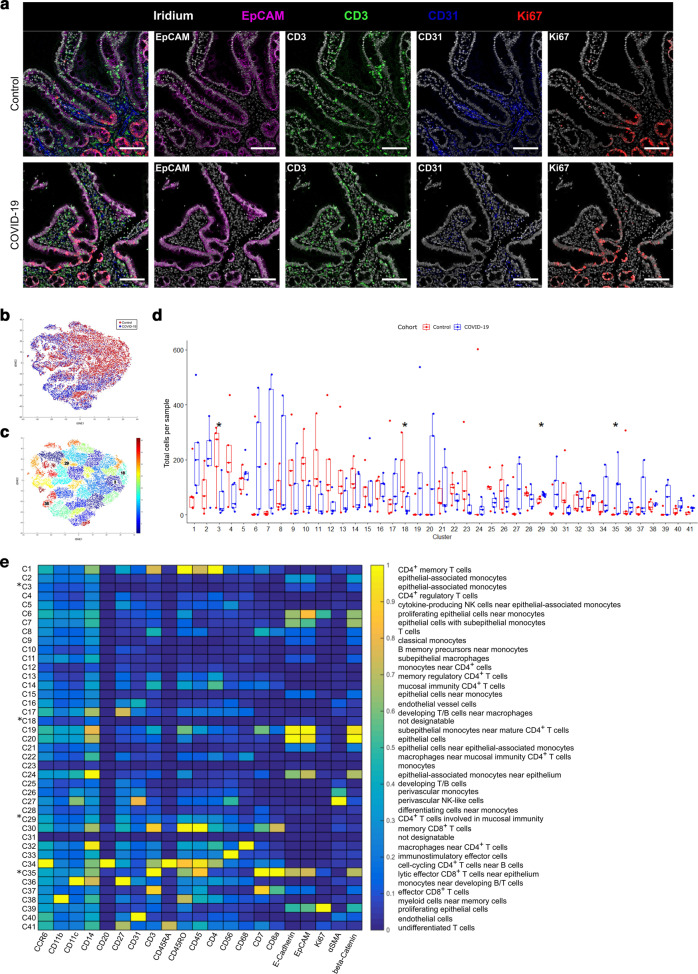
Small intestine reaction in COVID-19 patients is characterized by an epithelial increase of activated CD8^+^ T cells. **a** Representative images of imaging mass cytometry (IMC) of control (upper row, *n* = 5 biological replicates) and COVID-19 (lower row, *n* = 5 biological replicates) patients. Shown is the expression of EpCAM (magenta), CD3 (green), CD31 (blue) and Ki67 (red). Nuclei are counterstained with Iridium (grey). Scale bars represent 100 µm. **b** Overlay of tSNE plots of all segmented cells of all samples. COVID-19 patients are indicated in blue, control patients in red. **c** Phenograph clustering (k nearest neighbor = 80) of the generated tSNE plots. Colors indicate different clusters. Clusters marked with numbers show significant changes in cell abundance in cell numbers of COVID-19 patients in comparison to controls. **d** Box plot of total cell numbers per phenograph cluster per sample (red, control; blue, COVID-19). Four differently abundant clusters (*p* < 0.05) are marked with an asterisk (*). Boxes extend from 25–75th percentile, the line depicts the median. Dots represent single values of single samples. **e** Heatmap of mean expression per marker (*x*-axis) of each defined phenograph cluster (*y*-axis). Significantly different abundant clusters are marked with an asterisk. Cluster designations are given on the left of the heatmap according to their marker expression (Suppl. Table 4).

**Fig. 3:**
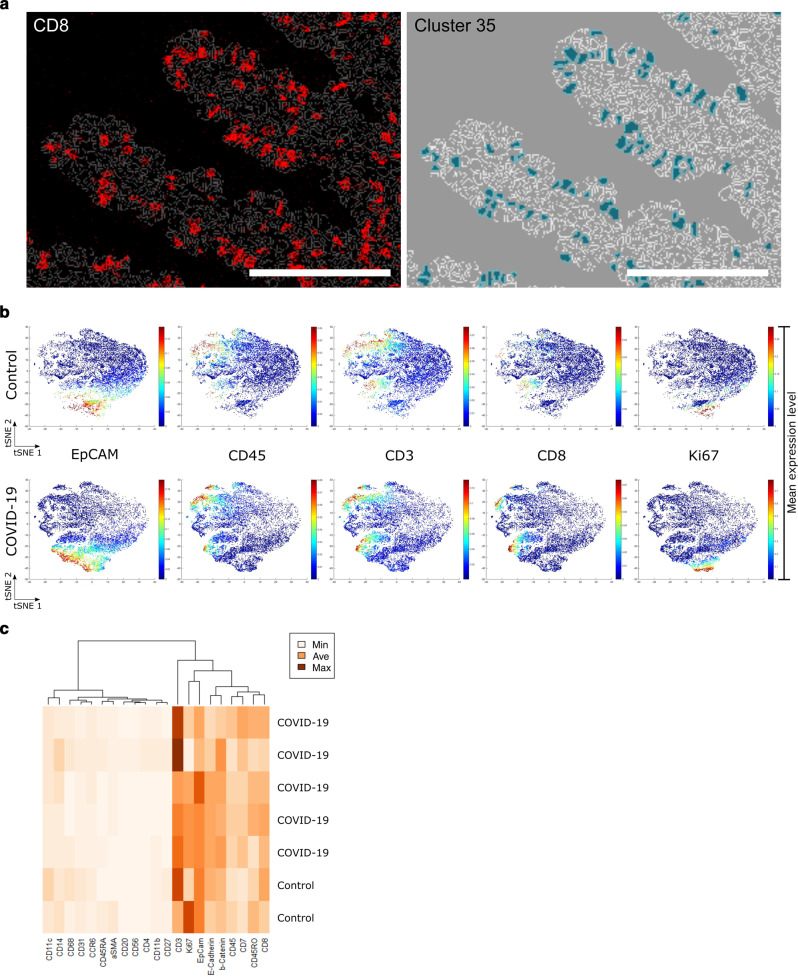
Analysis of Cluster 35 indicates IEL. **a** Imaging mass cytometry (IMC) image of CD8 staining (left) and cells designated to cluster 35 (right) overlaid with the computed cell masks. Scale bars represent 100 µm. **b** Single-cell tSNE maps show the expression of EpCAM, CD45, CD3, CD8, and Ki67. Plots show control (upper row) in comparison to COVID-19 (lower row) samples. The percentile cut-off was set to 99%. Color spectrum on the right of the plot indicates mean expression levels of the marker (red, high expression; blue, low expression). **c** Heatmap expression analysis of samples included in phenograph cluster 35 designated IEL. Marker expression levels columns are ordered according to hierarchical clustering. Dendrogram indicates similarity in marker expression. Color spectrum on the right of the plot indicates the mean expression levels of the marker (dark, high expression; light, low expression).

Biopsies of COVID-19 patients as well as of patients with peripheral arthralgia without any signs of gastrointestinal damage - the latter serving as controls were included in IMC analysis (*n* = 5 each). A higher abundance of CD3^+^ T cells in the epithelial layer of COVID-19 patients as well as a higher number of Ki67^+^ proliferating epithelial cells was observed, while no changes in CD31^+^ endothelial cells could be detected (Fig. 2a). For the processing and quantification of IMC data, cells were segmented by nucleus stains, to achieve an unbiased approach. To quantify the abundance of segmented cells, a tSNE dimensionality reduction algorithm served to visualize high dimensional single-cell data of 3,000–4,000 cells per sample (Fig. 2b). Due to the low signal-to-noise ratio of 147Sm (IL-13), 155Gd (FoxP3), 160Gd (T-bet), and 167Er (TNFα) channels, these markers were excluded from tSNE generation and Phenograph clustering (Suppl. Figures 2 and 3). An unsupervised Phenograph^28^ analysis was subsequently performed on the processed data, segmenting the cells into 41 clusters displaying different phenotypes (Fig. 2c). Four of these clusters (3, 18, 29, 30) were found to be differentially abundant in the COVID-19 cohort compared to controls (Fig. 2d). We found an increase in mucosal CD4^+^ T cells (cluster 29) (Fig. 2e and Suppl. Table 4) and an increase in CD8^+^ T cells within the epithelial layer, designated IEL (IEL, cluster 35) (Fig. 3a). As IEL are localized in between epithelial cells and the resolution of IMC is rather low, CD8^+^ IEL cluster within the epithelial cluster (Fig. 3b) supported by a weak expression aberrant of E-cadherin, EpCAM, and β-catenin in this cluster (Fig. 3c). As expected, the CD8^+^ IEL in this cluster are T cells (CD3^+^) of hematopoietic lineage (CD45^+^) and additionally express CD45RO and CD7, but not CD27 consistent with the phenotype of activated effector cells/antigen-experienced effector cells^29,30^. A low expression of Ki67 due to their proximity to Ki67^+^ epithelial cells was observed (Fig. 3c). In addition, COVID-19 patients presented with a non-significant increase of CD8^+^ T cell numbers within the lamina propria (LP) (cluster 30, *p* = 0.056) (Fig. 2d). Furthermore, the analysis revealed a slightly higher, non-significant abundance of CD4^+^ memory T cells in the LP (cluster 1, *p* = 0.056) of COVID-19 patients compared to control tissue.

We saw a significant decrease of CD14^+^ monocytes in the mucosal and subepithelial region in COVID-19 patients compared to controls (cluster 3). However, due to the up-regulation of epithelial markers in inflammation^31^, we suggest clusters 2 and 3 be the same cell type and therefore see no significant change in the abundance of monocytes. The IMC panel used in this study was designed to detect immunological changes in the duodenal mucosa and therefore did not include mesenchymal cell markers. This can be acknowledged in clusters 18, 23, and 31. Further studies will have to determine the mesenchymal changes in the duodenal mucosa of COVID-19 patients.

Naturally, resident intestinal macrophages are tolerant and express only low levels of CD14. The monocytes/macrophages detected in the duodenal mucosa by IMC revealed high expression of both CD68 and CD14 indicating their recent recruitment from the periphery (cluster 32) (Fig. 2e). Additionally, their expression of CD11b and CD11c was low indicating that they are already mature macrophages, while cluster 36 shows differentiating monocytes (CD14^+^ CD11c^hi^CD27^+^). There was no difference in cell numbers compared to control mucosa.

Taken together, our data demonstrate an accumulation of antigen-experienced activated intraepithelial CD8^+^ T cells in the small intestine of COVID-19 patients indicating anti-viral reactivity as a consequence of possible infection of the small intestinal epithelium by SARS-CoV-2.

To confirm these findings at a higher spatial resolution, multiplex immunohistochemistry was performed. Duodenal biopsies from COVID-19 patients (*n* = 5) and controls without intestinal damage (*n* = 9) were analyzed.

**Fig. 4:**
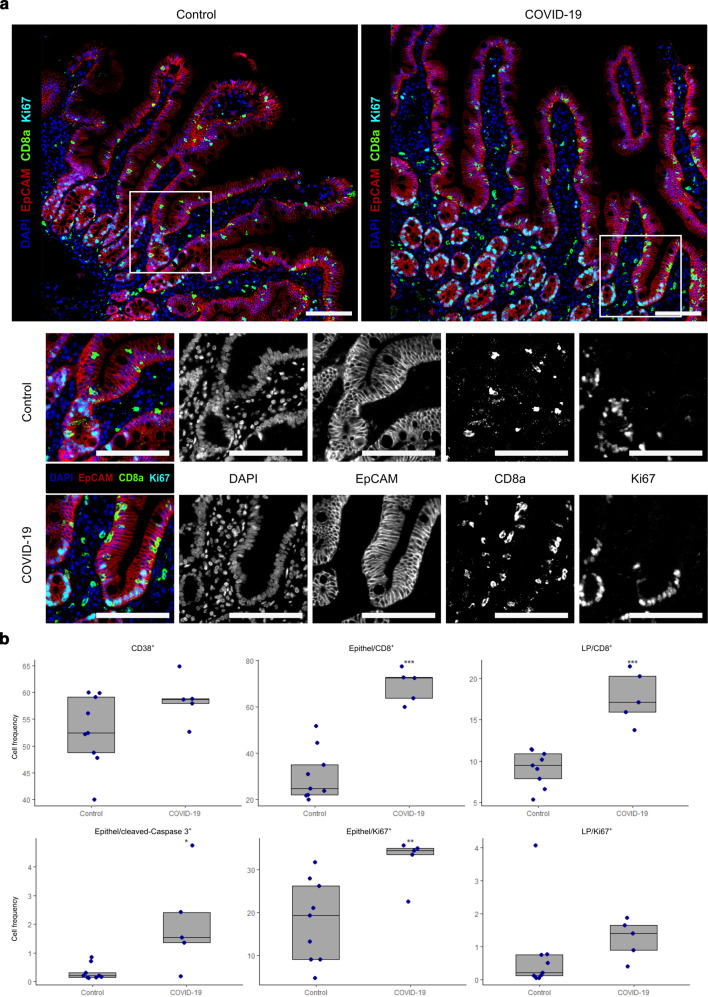
Multiplexing confirms imaging mass cytometry data and reveals epithelial apoptosis and regenerative response in COVID-19 patients. **a** Upper panel, representative images of immunostaining of EpCAM (red), CD8α (green), and Ki67 (cyan) on sections of control (left) and COVID-19 (right) patients. Nuclei are stained with DAPI (blue). Lower panels show magnifications of upper row pictures (white box) and single markers. Pictures on the left show merged colors, black and white pictures show individual markers. Note that Ki67^+^ epithelial cells are not restricted to the crypt compartment in COVID-19 but also in villus epithelium. Scale bars represent 100 µm. **b** Box plots of relative cell frequencies of CD38^+^ cells, CD8^+^ cells within the epithelium and CD8^+^ cells within the lamina propria (upper row) and cleaved caspase-3^+^ apoptotic epithelial cells, Ki67^+^ proliferating epithelial cells and Ki67^+^ proliferating cells within the lamina propria (lower row) of controls and patients with COVID-19. Significant differences in comparison to control samples (Wilcoxon signed-rank test) are marked with asterisks (*p < 0.05, **p < 0.01, ***p < 0.001). Boxes extend from 25–75th percentile, the line depicts the median. Dots represent individual data.

In multiplexing, the following antibody panels were used panel 1: antibodies directed against CD8α, EpCAM, Ki67, CD38; panel 2: EpCAM, Ki67, cleaved caspase-3; panel 3: EpCAM, CD8α, CD31, CD163, ACE2, TMPRSS2. Verifying the IMC data, multiplexing showed a higher number of CD8^+^ Ki67^−^ T cells within the epithelium of COVID-19 patients compared to controls (Fig. 4a and b). Furthermore, the numbers of Ki67^+^ proliferating epithelial cells were increased in COVID-19 patients compared to control patients indicating a regenerative response of the epithelium to the injury. For quantification, an unbiased automated tissue and cell segmentation approach, employing inForm software, was applied. The analysis indicated increased numbers of CD8^+^ cells within the epithelium of COVID-19 patients in comparison to control biopsies (69.4 ± 7.1 *vs.* 30.6 ± 11.2 cells per 100 epithelial cells (EC), *p* < 0.001), indicating a virus infection-induced infiltration. The numbers of CD8^+^ cells within the LP were increased in COVID-19 patients compared to controls (17.7 ± 3.2 *vs.* 9.1 ± 2.1 cells per 100 LP cells, *p* < 0.001) as well. Ki67^+^ proliferating epithelial cells were increased in COVID-19 patients compared to control patients (32.3 ± 5.5 *vs.* 18.1 ± 9.6 cells per 100 EC, *p* < 0.01), while the proliferation of lamina propria cells was comparable in COVID-19 patients and controls (1.2 ± 0.6 cells *vs.* 0.7 ± 1.3 cells per 100 LP cells). Cleaved caspase-3^+^ apoptotic epithelial cells were increased in COVID-19 patients compared to control patients (2.1 ± 1.7 *vs.* 0.3 ± 0.3 cells per 100 EC, *p* < 0.05). No differences in the number of cells expressing the late activation marker CD38 could be detected in COVID-19 patients in comparison to controls (51.2 ± 18.5 *vs.* 52.9 ± 6.7 cells per 100 LP cells).

Additionally, a smaller number of biopsies (COVID-19 *n* = 5, controls *n* = 6) were analyzed for the expression of ACE2 and TMPRSS2 as mediators of virus entry as well as for the expression of EpCAM, CD8α, CD31, and CD163. ACE2 was expressed on intestinal epithelial cells (Suppl. Figure 4A). TMPRSS2 expression was detected in intestinal epithelial cells as well as lamina propria mononuclear cells in all biopsies. There were no changes in CD163^+^ macrophage numbers or CD31^+^ endothelial cell numbers in COVID-19 patients (Suppl. Figure 4B) supporting the IMC data.

These findings confirm the data acquired by IMC at a higher spatial resolution. Furthermore, these data emphasize epithelial damage and subsequent regenerative processes possibly caused by an infection with SARS-CoV-2.

## Discussion

COVID-19 is not only a respiratory tract disease but also impairs the function and homeostasis of other organs. While some affected organs were tested positive for SARS-CoV-2^4–6^, others showed histopathological changes but did not show active viral replication as no SARS-CoV2 RNA could be detected within these organs^6,7,32^. Histopathological changes in these organs might result from an overreacting immune response^8^. As most of these histopathological findings were obtained from autopsies, there is a risk that tissues tested negative were already auto- or heterolytic and SARS-CoV-2 RNA may already have been degraded. In these cases, death may have occurred long after active virus replication. Additionally, the information about previous damage to the affected organs is limited. Furthermore, autopsy results were obtained from patients who had a severe and often long disease course of COVID-19 and the gastrointestinal mucosa is especially vulnerable to autolytic alteration. Thus, specimens from autopsies are not suitable for the evaluation of morphological changes of the small intestinal villi or the local immune response. The study presented herein focuses on COVID-19 patients with a mild to moderate disease course and on freshly collected tissue samples from the early phase of infection. As up to 17.6% of COVID-19 patients suffer from gastrointestinal symptoms^12^, the question arises whether these symptoms were induced by the virus targeting the gastrointestinal tract or as a consequence of a generalized activated and dysregulated immune system. To elucidate the gastrointestinal tract as a possible target and entry site for SARS-CoV-2, duodenal biopsies from COVID-19 patients were collected during esophagogastroduodenoscopy and compared to samples from patients without gastrointestinal damage.

The major transmission route of SARS-CoV-2 is through aerosols, droplets released into the air by the respiratory system of an infected person^33,34^. These droplets can be inhaled and infect the upper airways but can also be swallowed and pass the gastrointestinal tract. Thus, direct infection of epithelial cells could be speculated. In our cohort, SARS-CoV-2 RNA and nucleocapsid were demonstrated in 2 out of 5 specimens. Strikingly, we found the samples with the longest time between the onset of symptoms and esophagogastroduodenoscopy to be positive, regardless of the clinical symptoms. A similar percentage of positive samples has been found in a recently published study where viral nucleocapsid was found in 5 out of 14 patients at an average of 4 months after initial COVID-19 diagnosis^35^. While all of the patients investigated in this study were negative in nasopharyngeal swabs at the time of biopsy, three of the patients still showed a signal in PCR testing of the biopsies^35^. This is in contrast to another recently published study which detected SARS-CoV-2 nucleocapsid protein in small intestinal epithelial cells in 11 out of 12 COVID-19 patients tested at an average of 25 days after the last nasopharyngeal swab was tested positive^36^. In the same study, in eight out of 16 patients, viral particles could be detected in the epithelial cells of the duodenum and/or ileum. While no infectious virions were identified in the gastrointestinal tissue of patients suffering from COVID-19^36^, another study could prove the existence of infectious SARS-CoV-2 in feces of a single COVID-19 patient^37^. Altogether, it remains unclear at what time gastrointestinal infection by SARS-CoV-2 occurs and for how long it persists. In our study, the time or site of sampling might have missed the epithelial infection with SARS-CoV-2 in the biopsies, which tested negative for SARS-CoV-2.

**Fig. 5:**
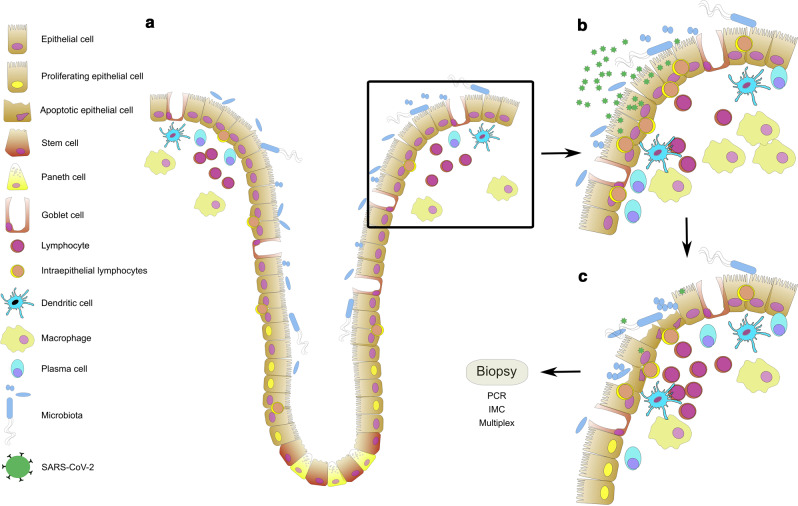
Schematic overview of the changes in the duodenal epithelium upon infection by SARS-CoV-2. **a** In health, homeostasis of the intestinal microbiota and the intestinal immune system is sustained. The intestinal epithelium serves as a barrier between the luminal microbiota and the mucosal immune system. It is a dynamic system in which proliferation, migration, differentiation, and apoptosis are balanced. The proliferation of stem and progenitor cells is confined to the crypt epithelium. **b** Upon infection of epithelial cells by SARS-CoV-2 the immune system gets activated: intraepithelial lymphocytes accumulate, cell numbers within lamina propria increase. **c** CD8^+^ IEL induces apoptosis in infected epithelial cells and the epithelial barrier breaks. Epithelial proliferation increases to repair the defects. Cellularity further increases within the lamina propria. Cells of the innate immune system clear the tissue from cell debris and pathogens. Cells of the adaptive immune system provide help and get activated. Mucosal homeostasis will be restored by an orchestrated immune reaction.

We here demonstrate that the most prominent changes in the duodenum of COVID-19 patients occur in the epithelium. In our study cohort, the average time from onset of symptoms to esophagogastroduodenoscopy was 8.2 days. At this time point, increased numbers of antigen-experienced activated CD8^+^ T cells were detected within the epithelium, indicating an enhanced migration of cells to the duodenal mucosa in the course of systemic SARS-CoV-2 infection. This is well in line with the results of a recent publication using mass cytometry to detect the immunophenotypic changes in the gastrointestinal mucosa of COVID-19 patients^36^. Surprisingly, even in this cohort, which consisted of patients on average 25 days after the last nasopharyngeal swab was tested positive, there was still an increase in CD8^+^ and CD8^+^ CD103^+^ T cells in the LP, but not within the epithelium. However, an increase in CD4^−^ CD8^−^ IELs could be observed. Although these IELs detected in our study most likely represent virus-specific CD8^+^ T cells, SARS-CoV-2 antigen specificity would need to be explored in future studies. In addition, duodenal biopsies displayed increased numbers of cleaved caspase-3^+^ apoptotic epithelial cells. One can speculate, that SARS-CoV-2 infected epithelial cells were killed by cytotoxic CD8^+^ T cells. These CD8^+^ T cells were characterized by low expression of Ki67 in IMC probably due to their close proximity to proliferating epithelial cells which expressed high levels of Ki67. In multiplexing, these CD8^+^ T cells were Ki67^-^. We assume an already foregone immune activation of CD8^+^ T cells including proliferation, which probably has occurred in lymphoid tissues followed by subsequent migration of activated CD8^+^ T cells to the mucosal target, similar to CD8^+^ T-cell activation and migration in HIV patients^38^. At the time point of biopsy, CD8^+^ IEL numbers were still high, but cells stopped proliferating (Fig. 5). Due to the low resolution of IMC, increased Ki67 expression of epithelial cells “spills over” into CD8^+^ IEL. Highly proliferating epithelial cells indicate a regenerative response to the injury. In contrast to the marked changes in the epithelial layer, the immune response within the lamina propria though seemed to be low, probably due to low levels of infection in intestinal tissues. This is on the one hand supported by histomorphology as well as by IMC. CD8^+^ lamina propria lymphocytes and CD4^+^ CD45RO^+^ T cells were slightly increased in numbers but showed no expression of Ki67 as a sign of proliferation in immunohistochemistry or IMC.

In blood samples from convalescent COVID-19 patients, CD8^+^ T cells were negative for various activation markers including CD38^39^. Similarly, CD8^+^ IEL did not express CD38 and CD27, but CD45RO and CD7. These recently activated CD45RO^+^ CD8^+^ IEL may play a role in clearing SARS-CoV-2 from the epithelium and in the resolution of COVID-19. Additionally, CD8^+^ T cells were also increased within the lamina propria. These differences may attribute to differences between periphery and tissue as well as to differences between active disease and convalescence.

A recent study, using an imaging mass cytometry approach on post-mortem tissue of three COVID-19 patients, demonstrated an increase in CD11b^+^ macrophage and CD11c^+^ dendritic cells^40^ in the intestine of COVID-19 patients. Though we did not detect any differences in CD163^+^ macrophage numbers in IHC or CD11b^+^ macrophage numbers in imaging mass cytometry, we did identify a higher abundance of CD11c^+^ CD14^+^ inflammatory/monocyte-derived dendritic cells (cluster 36) and a higher abundance of perivascular CD11c^+^ CD56^+^ cells (cluster 27).

In summary, we demonstrated the immunological response in the small intestinal epithelium following the systemic infection by SARS-CoV-2 in COVID-19 patients during the first two weeks after the onset of symptoms. To our knowledge, this study is the first study to describe the early immunological changes in the small intestinal mucosa early after the onset of COVID-19. We hypothesize the migration of CD8^+^ T cells to the epithelial cell layer to be the early consequence of the possible epithelial infection by SARS-CoV-2. However, one could also imagine these immunological changes to be the consequence of a general immune activation mediated by the SARS-CoV-2 infection of the lung, since the infection of the intestinal mucosa seems to be weak. Consequently, gastrointestinal symptoms could be of other consequence than direct infection of the intestinal mucosal tissue by SARS-CoV-2. The possible early infection seems to be mainly characterized by a migration of activated CD8^+^ IEL to the duodenal mucosa (Fig. 5a and b) and consecutive villous blunting characterized by apoptosis followed by regeneration (Fig. 5c). Although histomorphologic changes seem to be the only minor in the duodenal mucosa and the study presented herein is limited by sample numbers, the clinical significance of possible infection of the gastrointestinal tract by SARS-CoV-2 still needs to be evaluated since, until now, there is contradicting data concerning the significance of gastrointestinal symptoms for the disease progression^36,41–43^. Thus, long-term effects as well as a possible SARS-CoV-2 infection of other parts of the gastrointestinal system have still to be evaluated.

## Materials and methods

### Patients and controls

COVID-19 patients were recruited from April to June 2020. Five COVID-19 patients were included in this study, presenting with either upper abdominal pain or diarrhea. Clinical information is provided in Table 1. During esophagogastroduodenoscopy, a total of four duodenal biopsies were taken of each patient. As controls, archived biopsies of patients with peripheral arthralgia without any signs of gastrointestinal damage (*n* = 9, 49–85 years, 4 female, 5 male) were included (Suppl. Table 1). In COVID-19 patients, SARS-CoV-2 infection was confirmed via qRT-PCR from nasopharyngeal swabs as described below. The study was approved by the ethics committee of the Charité-Universitätsmedizin Berlin (Nr. EA2/072/20) and all patients gave written consent.

### RNA isolation and quantitative real-time PCR

SARS-CoV-2 infection was confirmed via qRT-PCR from nasopharyngeal swabs as described before^44,45^. For SARS-CoV-2 RNA detection, unfixed biopsy tissue samples, stored in 0.9% NaCl were used. RNA was extracted by using the MagNAPure 96 system and the MagNAPure 96 DNA and Viral NA Large Volume Kit (Roche, Basel, Switzerland) according to the manufacturer’s instructions. In the case of SARS-CoV-2 detection, quantification of SARS-CoV-2 RNA was performed using serial diluted quantified in vitro RNA transcripts, as described previously^46^. SARS-CoV-2 RNA concentrations are given as viral RNA copies per 10,000 cells, assuming 1 ng DNA is representing 152 diploid cells. DNA was measured by using the Qubit dsDNA HS Assay kit (Thermo Fisher Scientific, Waltham, MA, USA).

### Cell culture

Vero-E6 cells were cultivated in DMEM (PAN-Biotech, Aidenbach, Germany) medium supplemented with 2% fetal calf serum and 100 units/ml of Penicillin and 0,1 mg/ml Streptomycin (PAN). At a confluence of 60–80%, cells were infected with SARS-CoV-2 at a multiplicity of infection of approximately 0.02. Fourty-eight hours later, the cytopathic effect was pronounced and cells were harvested at 90–100% confluency with trypsin-EDTA (PAN, 0.05% trypsin, and 0.02% EDTA) and washed twice. Vero-E6 cells cultured in the absence of virus served as mock control. Cell pellets were overlaid with an around 10-fold volume of 10% neutral buffered formalin (Roti^®^Histofix 10%, Roth, Karlsruhe, Germany) and stored at 4 °C for at least 24 h before embedding.

### Histopathology

Biopsies taken during esophagogastroduodenoscopy were transported on 0.9% NaCl to the lab and transferred to 10% formalin (SAV Liquid Production, Flintsbach a. Inn, Germany) within 30 min. Samples were fixed overnight at room temperature and embedded in paraffin (Histosec, Merck, Darmstadt, Germany) the next day. Paraffin blocks were prepared, and sections were freshly cut for histochemistry and immunofluorescence (1–2 µm) as well as imaging mass cytometry (IMC) (4 µm). Archived samples consisted of paraffin blocks. This material underwent similar processing. For the evaluation of histomorphology, paraffin sections were dewaxed, histochemically stained with hematoxylin (Merck, Munich, Germany) and eosin (Merck), and coverslipped with Histokitt (Roth).

### Imaging mass cytometry (IMC)

For IMC, biopsies of COVID-19 patients (*n* = 5) and controls (*n* = 5) were used. Paraffin sections were dewaxed prior to heat-induced antigen retrieval at pH 6.0 followed by a blocking step in 3% BSA (Albumin Bovine Fraction V, Serva, Heidelberg, Germany) in antibody diluent (Agilent, Santa Clara, USA). Sections were incubated overnight at 4 °C with a cocktail of metal-conjugated antibodies (Suppl. Table 2) prepared in 0.5% BSA (Serva)-containing antibody diluent (Agilent). Pre-labeled antibodies were purchased from Fluidigm (San Francisco, USA). Unlabeled antibodies were purchased in the carrier-free buffer as indicated in Suppl. Table 2 and labeled using MaxPar Antibody X8 conjugation kits (Fluidigm) according to the manufacturer’s instructions and reconstituted in PBS-based antibody stabilizer (CANDOR Bioscience, Wangen, Germany) at a concentration of 0.5 mg/ml. Nuclei were stained with CELL-ID Intercalator-Ir (1:400, Fluidigm) in antibody diluent (Agilent). Slides were rinsed, air-dried, and stored at room temperature until measurement.

For IMC data acquisition, a CyTOF2/upgraded to Helios specifications coupled to a Hyperion Tissue Imager (Fluidigm) was used, using the CyTOF software version 6.5.236. Prior to sample ablation, the instrument was tuned according to the manufactures instructions, using the 3-Element Full Coverage Tuning Slide (Fluidigm). The dried slide was loaded into the imaging module and regions of interest were selected for each sample on a preview of the sample. Optimal laser power was determined for each sample to obtain complete ablation of the tissue. Laser ablation was performed at a resolution of 1 µm and a frequency of 200 Hz. Data were stored as mcd-files as well as txt-files. Signal spillover between channels was accounted for by using the CATALYST R package as described^47^.

Single-cell analysis was done using the IMC segmentation pipeline as published^48^. In short, the imc_preprocessing python script was used to convert the MCD and txt files, followed by a CellProfiler (version 3.1.9)^49^ pipeline for cropping and up-scaling the images for further analysis. A pixel classifier was trained for the classification of nuclei, cytoplasm, and background using Ilastik (version 1.3.3rc2)^50^. Thus, the iridium channels were used for constructing probability maps with constant checking for uncertainties. The created probability maps were used in CellProfiler to create single-cell masks. Each of the cell masks was transferred according to TIFF files into HistoCAT (version 1.7.1)^51^ for analysis of cell phenotypes. HistoCAT software was first used for an arcsinh transformation (cofactor = 5) of the data. An area containing 3,000–4,000 cells was gated based on optimal overall staining to analyze comparable cell numbers per sample. A dimensionality reduction tSNE algorithm^52^ was used for the visualization of all single-cell data, excluding 147Sm (IL-13), 155Gd (FoxP3), 160Gd (T-Bet), and 167Er (TNFα) due to high background staining. For cluster analysis, the Phenograph^28^ function of HistoCAT served to cluster cells according to their marker expression (k nearest neighbor = 80). The resulting data were exported for statistical analysis using HistoCAT.

### Immunofluorescence

For multiplexing, paraffin sections were dewaxed prior to heat-induced antigen retrieval at pH 6.0. Endogenous peroxidase was blocked with Dako REAL Peroxidase-Blocking Solution (Agilent) prior to incubation with the first primary antibody followed by incubation with peroxidase-labelled secondary antibody employing the EnVision+ HRP Labelled Polymer EnVision system (Agilent). For detection, the OPAL system was used (Akoya Biosciences, Menlo Park, USA). Before starting the second round of staining with primary and secondary antibodies, proteins and enzymes were inactivated by heat and acidic pH. A total of three, four, and six, respectively, staining cycles employed the following primary antibodies: Panel 1: CD8, CD38, Ki67, EpCAM; Panel 2: CD8, EpCAM, cleaved caspase-3; Panel3: CD163, CD31, ACE2, TMPRSS2, CD8, EpCAM (Suppl. Table 3). After the last inactivation step (in order to get rid of doublets), nuclei were stained using 4′,6-diamidine-2′-phenylindole dihydrochloride (DAPI, Akoya Biosciences) and sections were coverslipped using Fluoromount G (Southern Biotech, Birmingham, USA). For SARS-CoV-2 nucleocapsid staining, the same technique was used with a SARS/SARS-CoV-2 Coronavirus nucleocapsid monoclonal antibody (clone B46F, ThermoFisher Scientific) employing the OPAL system as mentioned above. Vero cells infected with SARS-CoV-2 for 48 h served as a positive control. Pictures were acquired via the Vectra 3 Automated Quantitative Pathology Imaging System (Akoya Bioscience) employing Vectra3 (Akoya Bioscience, version 3.0.7) and Phenochart (Akoya Bioscience, version 1.0.12). A total of five (*n* = 5) multispectral images (MSI) for every biopsy of a patient (*n* = 3) were acquired (*n* total = 15) using the DAPI and the EpCAM staining for an overview. For analysis, single-cell information was exported using the inForm software (Akoya Bioscience, version 2.4.8) and analyzed for cell frequencies using R (version 4.0.2) and RStudio (version 1.3.959) employing the phenoptr and phenoptrReports (version 0.2.8) packages^53^. Image processing for visualization of single markers was done using ImageJ (version 1.53a).

### Statistical analysis

Data processing and analysis as well as statistical testing was carried out in an unsupervised manner. Statistical analysis and visualization were done using R (version 4.0.2) and RStudio (version 1.3.959). Statistical significance was calculated using Student’s *t*-test and Wilcoxon signed-rank test, respectively. Statistical significance was considered for *p* < 0.05.

## Supplementary information

Supplementry Material

Supplementry Material
