# Supplementary material for: Human small intestinal infection by SARS-CoV-2 is characterized by a mucosal infiltration with activated CD8+ T cells
Source: Mucosal Immunol. 2021 Aug 21;14(6):1381–92. doi: 10.1038/s41385-021-00437-z (PMC8379580; doi:10.1038/s41385-021-00437-z)
Supplement: Supplementary file 2 — Supplementry Material [file 41385_2021_437_MOESM2_ESM.pdf]

Supplementary material

Suppl. Figure 1

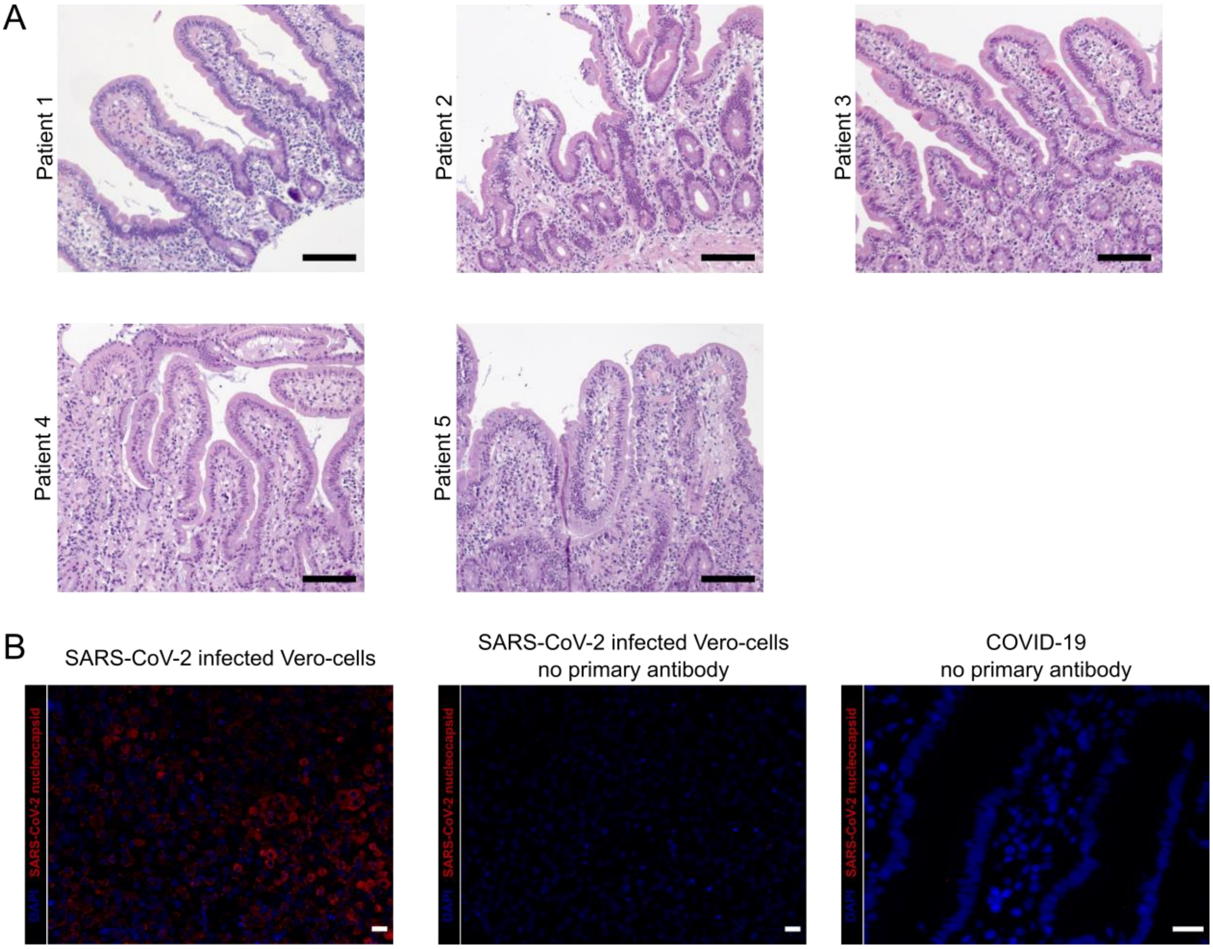

Suppl. Figure 1: Representative images of duodenal biopsies of COVID-19 patients and controls for immunohistochemical staining of SARS-CoV-2 nucleocapsid (**A**) Representative images of H&E staining of duodenal biopsies acquired from patients suffering from COVID-19. Scale bars represent 100 µm. (**B**) Representative images of SARS-CoV-2 nucleocapsid (red) and DAPI (blue) staining of Vero-cells 48 h after infection with SARS-CoV-2 with (left) and without primary antibody (middle) and control of SARS-CoV-2 nucleocapsid (red) and DAPI (blue) staining of a duodenal biopsy from a COVID-19 patient with no primary antibody (right). Scale bars represent 20 µm.

Suppl. Figure 2

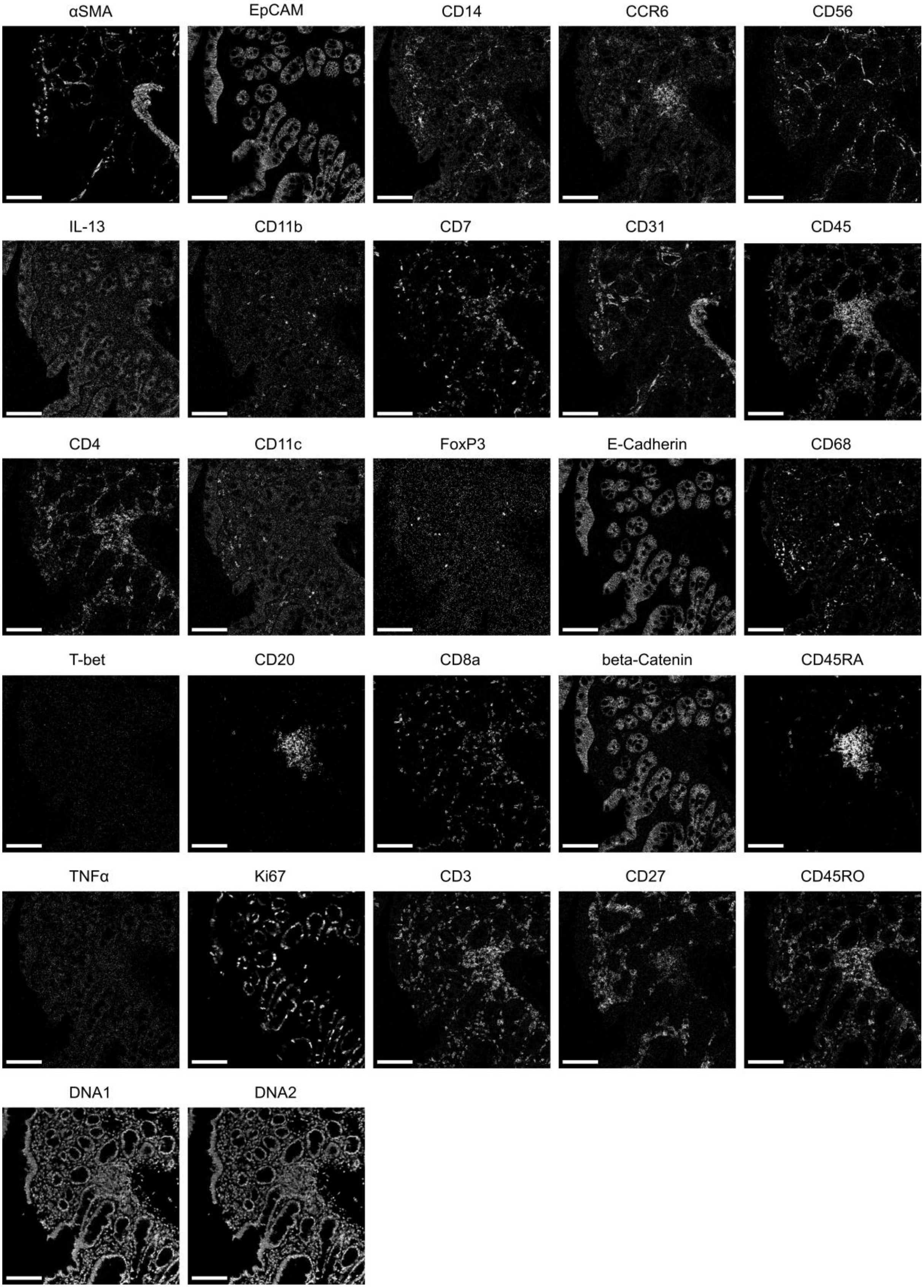

Suppl. Figure 2: Representative images of each marker used for imaging mass cytometry.

The images show representative staining of each marker used in imaging mass cytometry.

Brightness and contrast were adjusted for each image individually. Scale bars represent 100 μm.

Suppl. Figure 3

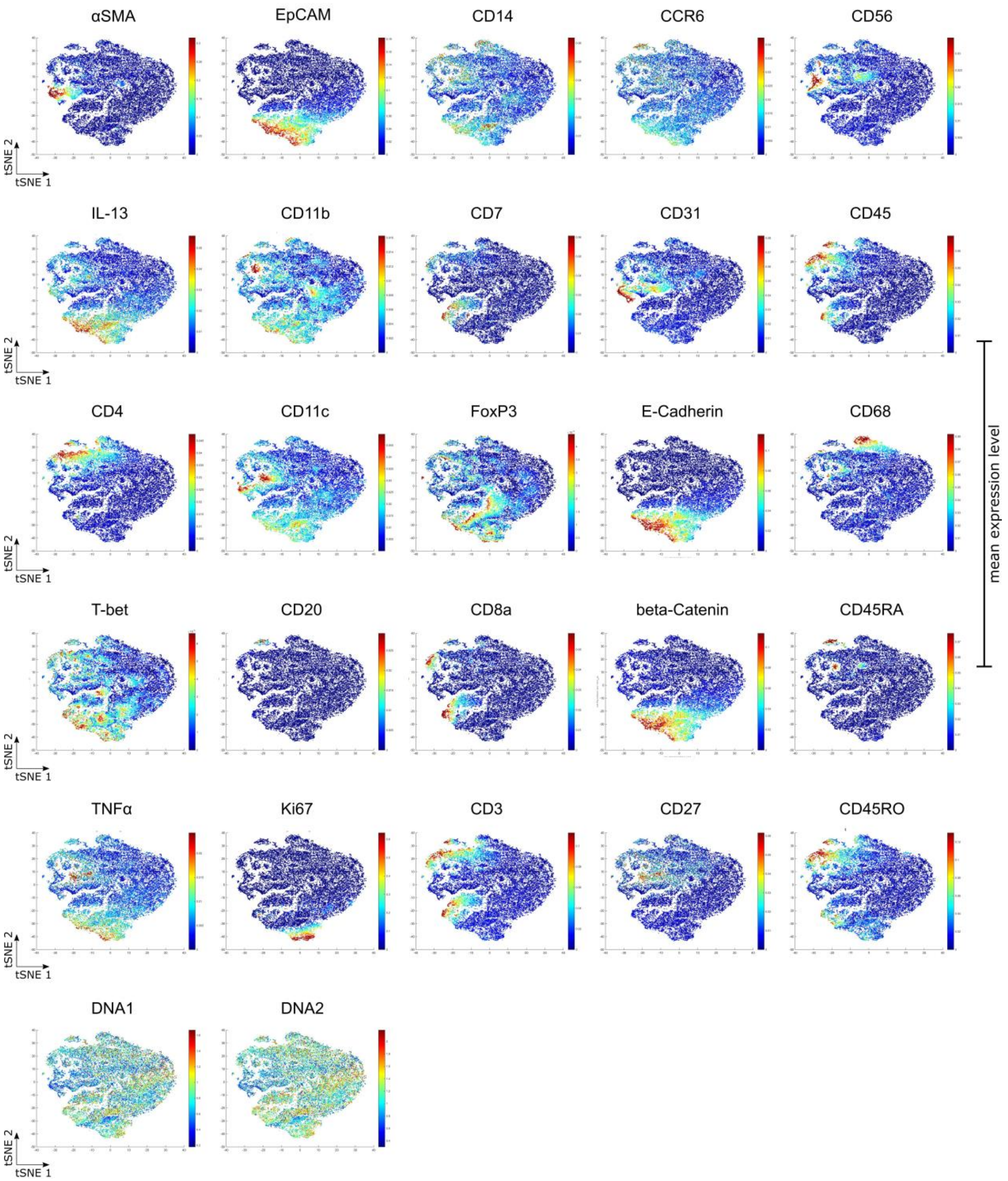

Suppl. Figure 3: tSNE plot of overall cells overlaid with heatmaps of the respective marker. The figure shows tSNE plots of all cells overlaid with individual heatmap of each marker measured. Cut-off was set to the 99% percentile. Color spectrum on the left of the plot indicates mean expression levels of the marker (red, high expression; blue, low expression).

Suppl. Figure 4

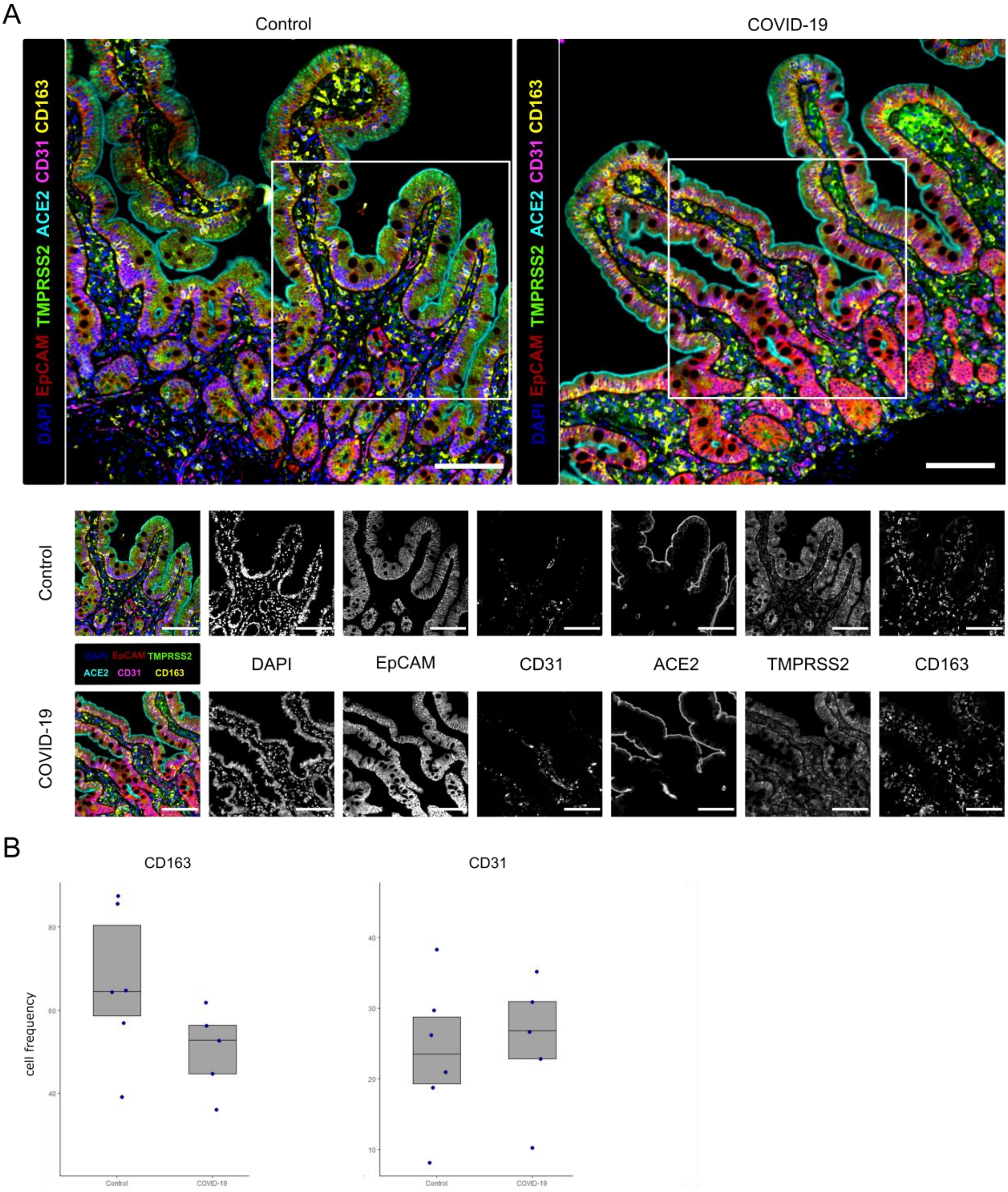

Suppl. Figure 4: Multiplexing shows expression of SARS-CoV-2 entry proteins on small intestinal enterocytes of COVID-19 patients and no changes in CD31⁺ and CD163⁺ cells. (**A**) Upper row, representative images of immunostaining of EpCAM (red), CD31 (magenta), ACE2 (cyan), TMPRSS2 (green) and CD163 (yellow) and nuclei (DAPI, blue) on sections of control (left) and COVID-19 (right) patients. The color for the respective marker is shown on the left of the image. Lower panels show extracts of upper row pictures (white box) and

single markers. Pictures on the left show merged colors, black and white pictures show individual markers. Scale bars represent 100 µm. (**B**) Box plots of relative cell frequencies of $CD163^{+}$ macrophages and $CD31^{+}$ endothelial cells within the lamina propria. No significant differences in relative cell frequencies could be observed. Boxes extend from 25th to 75th percentile, the line depicts the median. Dots represent individual data.

Suppl. Table 1: Control patients

Control patients	
Age	Sex
85	M
79	M
52	F
62	F
75	M
77	F
61	M
49	M
80	F

Suppl. Table 2: Antibodies used for imaging mass cytometry

isotope tag	target	clone / company	catalogue number	dilution
^{141}Pr	αSMA	1A4 / Fluidigm	3144047D	1:2000
^{143}Nd	EpCAM	E6V8Y / Cell Signaling Technologies	93790	1:2000
^{144}Nd	CD14	EPR3653 / Fluidigm	3144025D	1:400
^{145}Nd	CCR6	EPR22259 / Abcam	243852	1:200
^{146}Nd	CD56	E7X9M / Cell Signaling Technologies	99756	1:200
^{147}Sm	IL-13	polyclonal / Abcam	9576	1:200
^{149}Sm	CD11b	EPR1344 / Fluidigm	3149028D	1:200
^{150}Nd	CD7	EPR4242 / Abcam	243852	1:800
^{151}Eu	CD31	EPR3094 / Fluidigm	3151025D	1:100
^{152}Sm	CD45	D9M8I / Fluidigm	3152018D	1:4000
^{153}Eu	CD4	EPR6855 / Fluidigm	181724	1:400
^{154}Sm	CD11c	polyclonal / Fluidigm	3154025D	1:100
^{155}Gd	FoxP3	236A/E7 / Fluidigm	3155016D	1:400
^{158}Gd	E-Cadherin	24E19 / Fluidigm	3158029D	1:2000
^{159}Tb	CD68	KP1 / Fluidigm	3159035D	1:800
^{160}Gd	T-Bet	4B10 / BioLegend	644802	1:200
^{161}Dy	CD20	H1 / Fluidigm	3161029D	1:2000
^{162}Dy	CD8a	C8/144B / Fluidigm	3162034D	1:1500
^{165}Ho	β-Catenin	D13A1 / Fluidigm	3165032D	1:2000
^{166}Er	CD45RA	HI100 / Fluidigm	3166028D	1:1000
^{167}Er	TNFα	M1-C4 / Sigma-Aldrich	SAB1404480	1:400
^{168}Er	Ki67	B56 / Fluidigm	3168022D	1:1000
^{169}Tm	HistonH3	D1H2 / Cell Signaling Technologies	114499	1:2000
^{170}Er	CD3	polyclonal / Fluidigm	3170019D	1:50
^{171}Yb	CD27	LG.3A10 / Fluidigm	3171025D	1:200
^{173}Yb	CD45RO	T200/797 / Abcam	212786	1:1500
^{191}Ir	DNA	Fluidigm	201192A	1:400
^{193}Ir	DNA	Fluidigm	201192A	1:400

Suppl. Table 3: Antibodies used for multiplex immunohistochemistry

OPAL	target	clone	company	catalogue number	dilution
			Panel 1		
570	CD8	C8/144B	Agilent	IS623	1:30
650	CD38	E7Z8C	Cell Signaling Technologies	51000	1:50.000
690	Ki67	MIB1	Agilent	M7240	1:2.000
520	EpCAM	E6V8Y	Cell Signaling Technologies	93790	1:4.000
			Panel 2		
650	cleaved caspase-3	Asp175	Cell Signaling Technologies	9661	1:1.500
520	EpCAM	E6V8Y	Cell Signaling Technologies	93790	1:4.000
570	CD8	C8/144B	Agilent	IS623	1:30
			Panel 3		
540	CD163	10D6	Novocastra/Leica	NCL-L-CD163	1:2.000
620	CD31	89C2	Cell Signaling Technologies	3528	1:2.000
690	ACE2	EPR4435(2)	Abcam	ab108252	1:4.000
570	TMPRSS2	EPR3861	Abcam	ab92323	1:50.000
520	CD8	C8/144B	Agilent	IS623	1:30
650	EpCAM	E6V8Y	Cell Signaling Technologies	93790	1:4.000

Suppl. Table 4: Designation of phenograph clusters by marker expression
